# Shiga Toxin–producing *Escherichia coli* Serogroups in Food and Patients, Germany

**DOI:** 10.3201/eid1411.080361

**Published:** 2008-11

**Authors:** Dirk Werber, Lothar Beutin, Rohtraud Pichner, Klaus Stark, Angelika Fruth

**Affiliations:** Robert Koch Institute, Berlin, Germany (D. Werber, K. Stark, A. Fruth); Federal Institute for Risk Assessment, Berlin (L. Beutin); Max-Rubner Institute, Kulmbach, Germany (R. Pichner)

**Keywords:** Escherichia coli O157, Shiga toxin, food, food poisoning, public health, diarrhea, dispatch

## Abstract

We compared 61 Shiga toxin–producing *Escherichia coli* (STEC) serogroups from 448 food isolates with 71 STEC serogroups from 1,447 isolates from patients in Germany. Two thirds (41/61), representing 72% of food isolates, were also found in patients. Serogroups typically isolated from patients with hemolytic uremic syndrome were rarely found in food.

Shiga toxin–producing *Escherichia coli* (STEC) of serogroups other than O157 (non-O157 STEC) account for 80% of STEC gastroenteritis reports in Germany’s national surveillance database (*1*). Some of the non-O157 serogroups unequivocally cause disease comparable in severity to that caused by STEC O157, such as the hemolytic uremic syndrome (HUS) (*2*). Numerous, but not all, STEC serogroups have been linked with human disease.

Food is an important transmission vehicle for human STEC infection, especially in outbreaks (*3*), and many different STEC serogroups are isolated from food (*4*). Yet the public health relevance of many of these STEC serogroups, which includes their ability to cause human disease and the frequency with which this may occur, has not been investigated.

In Germany, identification of STEC in patients’ stool and in food is based on detection of Shiga toxin or of a Shiga toxin gene and subsequent isolation of STEC strains (*4*,*5*). This allows, in principle, ascertainment of all STEC strains, independent of their serogroup. To assess the public health relevance of STEC isolated from food, we compared those strains with those isolated from patients.

## The Study

Information on STEC isolates from food came from 2 sources. The first source was the Federal Institute for Risk Assessment, which received isolates from German governmental food inspection laboratories for strain characterization from 2005 through 2007 (food source 1). These STEC isolates originated from routine food samples taken by food safety authorities across Germany, according to a nationwide sampling scheme that focused during the sampling period mainly on red meat, ground raw meat, and stabilized meat products. The second source was the Max-Rubner Institute in Germany, which had conducted a series of investigations in conveniently selected meat-processing companies in Germany from 1996 through 2004 (food source 2). Information on STEC isolates from patients came from a laboratory-based sentinel in existence from 1999 through 2004, coordinated by Germany’s National Reference Center. The sentinel has been described elsewhere (*6*). In brief, private laboratories across Germany agreed to screen stool specimens of gastroenteritis patients for the presence of Shiga toxin 1 and Shiga toxin 2 with an enzyme immunoassay if predefined criteria were met (e.g., patients with diarrhea were <5 years of age, bloody diarrhea was mentioned on the laboratory request form). Positive samples were sent to the National Reference Center, where STEC strains were isolated and subtyped by various methods (including serotyping).

We calculated frequencies and proportions of STEC serogroups separately for food and patient isolates. Serogroups were compared for matches in both groups. Because the clinical outcome associated with human STEC infection was not systematically recorded, we additionally compared serogroups of food isolates with a compilation (available on the Internet) of literature reports of STEC serotypes and their association with human illness (*7*). We acknowledged an association with human illness if a symptom at least as severe as diarrhea was specified for a serogroup. The proportion of serogroups in patient and food isolates was compared by using the Wilcoxon signed rank test. Within selected serogroups, we examined serovars (classified by O and H antigen, e.g., O157:H7) to assess comparability between food and patient isolates because the serovar is a better proxy for genomic background of the strains than is the serogroup.

Serogroup information for STEC isolated from food was available for 448 strains (including nontypeable strains [Ont] and self-agglutinating isolates [Orough]), 357 from food source 1 and 91 from food source 2 (Table 1). The most common of the 61 serogroups identified in food isolates were O8 (9%), O91 (6%), and O113 (5%) (Table 2). Commonalities, but also differences, were observed between the food sources. For example, the proportion of serogroups O8 and O91 was high in both food sources, whereas O113 strains were isolated only from food source 1. Notably, STEC isolates from game represented 24% (85/357) of strains isolated in governmental food inspection laboratories (Table 1). Game also had the highest STEC prevalence among the different food categories routinely sampled by Germany’s food safety authorities from 2005 through 2006 (13%, 95% confidence interval 9%–17%) (*8*,*9*). Serogroup information for STEC isolates from patients was available for 1,447 of 1,478 (including Ont and Orough). Overall, 71 different STEC serogroups were isolated, and O157 (18%) was the most frequently serotyped O-group, followed by O103 and O26 (14% each; Table 2). No secular trends were observed during the study period, but proportions of single serogroups varied across years, particularly for STEC O103 isolates (range 6%–24%).

**Table 1:** Foods from which STEC was isolated in Germany, 1996–2004*

Source	Origin	No. ruminant samples†	Total no. samples
Federal Institute for Risk Assessment‡	Ground beef§	112	112
	Game¶	57	85
	Raw milk	55	55
	Beef and beef products	43	43
	Raw spreadable sausages	20	20
	Pork and pork products	0	19
	Cheese	15	15
	Other#	2	7
Max-Rubner Institute**	Raw spreadable sausages	54	54
	Raw sausage meat	34	34
	Other††	2	3
Total		395	448

*STEC, Shiga toxin–producing *Escherichia coli*. †Samples from food containing ruminant meat or of ruminant origin. ‡Isolates from governmental food inspection laboratories (2005–2007). §Of these, 12 contained also ground meat from sheep. ¶Deer (n = 56), wild boar (n = 18), hare (n = 10), game unspecified (n = 1). #Sheep meat (n = 2), duck, salad, flan, minced horse meat, river water (1 each). **Isolates obtained during a series of investigations in selected meat-processing companies (1996–2004). ††Meat juice (n = 2), horse meat (n = 1).

**Table 2:** Serogroups of STEC most frequently isolated from patients (1999–2004) and food (1996–2007), Germany*

Frequency ranking	Serogroups isolated from patients (%)	Serogroups isolated from food† (%)
1	O157 (18)	O8 (9)
2	O103 (14)	O91 (6)
3	O26 (14)	O113 (5)
4	O91 (10)	O22 (4)
5	O145 (4)	O115 (4)
Total percentage‡	60	28

*STEC, Shiga toxin–producing *Escherichia coli*. †Food categories from which strains were isolated: O8: raw spreadable sausage (n = 14), pork and products (n = 9), ground beef (n = 7), raw sausage meat (n = 5), raw milk (n = 4), game (n = 2), other (n = 1). O91: raw spreadable sausage (n = 14), ground beef (n = 5), raw milk (n = 3), game (n = 2), raw sausage meat (n = 2), other (n = 2). O113: ground beef (n = 10), cheese (n = 3), game (n = 3), pork and products (n = 2), beef and products thereof (n = 1), raw milk (n = 1), raw spreadable sausage (n = 1). O22: ground beef (n = 8), raw spreadable sausage (n = 6), beef and beef products (n = 1), game (n = 1), ground beef (n = 1), raw milk (n = 1), raw sausage meat (n = 1). O115: raw sausage meat (n = 12), raw spreadable sausage (n = 3). ‡Percentage of all isolates for which serogroup information was available (1,447 isolates from patients and 448 isolates from food).

Of the 61 food serogroups, 41 (67%) were also identified in patients (Figure). These serogroups comprised 72% (242/339) of food isolates with a known serogroup. Similarly, 78% (19/25) of serogroups isolated from game, accounting for 70% (44/63) of isolates, occurred also in patients. The Internet search showed a published association with human illness for at least 41 (67%) of all food serogroups; the phrase “at least” is used because 5 serogroups (O174, O176-O179) were officially acknowledged as genuine O-groups after May 2003 (*10*), which according to the website is the date of its last update (*7*). Moreover, some serogroups exclusively found in food in this study (and not listed on the website) have been described as sporadic patient isolates from Germany (*11*) and elsewhere (*12*).

**Figure:**
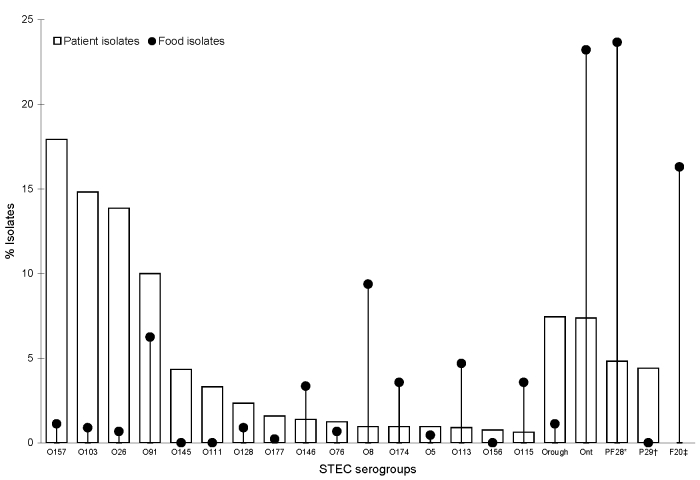
Proportion of Shiga toxin–producing *Escherichia coli* (STEC) serogroups identified in patients during a laboratory-based sentinel surveillance program (1999–2004) compared with STEC from food (1996–2004), Germany. *Comprises 28 serogroups that each accounted for <0.5% of patient isolates and were also identified in food isolates (O2, O4, O6, O15, O22, O23, O30, O38, O40, O55, O74, O84, O87, O88, O101, O102, O104, O110, O112, O119, O120, O121, O136, O148, O163, O171, O178, O179). †Comprises 29 serogroups that each accounted for <0.5% of patient isolates but were not identified in food isolates (O1, O7, O9, O12, O14, O17, O18, O25, O51, O60, O69, O70, O71, O75, O77, O78, O80, O86, O90, O93, O98, O106, O117, O118, O150, O154, O165, O167, O181). ‡Comprises 20 serogroups identified in food isolates only (O11, O21, O27, O36, O46, O56 O59, O62, O79, O82, O100, O109, O116, O126, O130, O141, O153, O166, O172, O176).

Overall, a significant inverse correlation was found between the ranking of the serogroup proportion in patients and in food (p<0.01). This finding is illustrated by the following: of the 41 serogroups found in food and in patients, 33 accounted each for <1% of the patient isolates. In total, they represented only 9% of patient isolates but 45% of food isolates. Conversely, the 3 most frequently identified serogroups in patients, O157, O103, and O26, represented 46% of the patient isolates but only 3% of food isolates. These 3 serogroups account for 85% of STEC isolated in pediatric HUS patients in Germany (*2*). Notably, the virulent serogroup O157 was found in 5 (1%) food isolates. This result is compatible with results of studies conducted in other countries that identified only few, if any, O157 strains among STEC strains isolated from ruminant meat, particularly beef (*13*,*14*).

At least 1 serogroup, O91, was frequently isolated from both food and patients. In food, O91 strains were the second (6%), and in patients the fourth (10%), most commonly identified O-group (and the most commonly identified O-group in adults; data not shown). On the serovar level, a comparable distribution was observed between isolates from patients and food: in both groups, the same 3 serovars (O91:H–, O91:H14, O91:H21) could be distinguished; O91:H– was the most common STEC serovar in patient isolates (64%, 94/145) as well as in food isolates (47%, 19/41). A different situation was observed for STEC O113, the third (5%) most frequently serotyped O-group in food. Among patient isolates, STEC O113 was not that frequently isolated: the serogroup ranked 14th and comprised 1% of patient isolates. Furthermore, a greater heterogeneity between patient and food isolates was found in O113 strains. Although the same 3 serovars were identified in both groups (O113:H–, O113:H4, O113:H21), O113:H4 was the predominant STEC serovar in isolates from patients (70%, 9/13), whereas O113:H21 (81%, 17/21) was the most common in food isolates. The latter serovar is frequently isolated from nonpediatric HUS patients (*15*).

## Conclusions

Two thirds (41/61) of serogroups from food were also isolated from patients, comprising 72% of food isolates with a known serogroup. These serogroups included, albeit uncommonly, those typically identified in pediatric and nonpediatric HUS patients. An association with human illness has been published for more than two thirds of food serogroups. These findings suggest that many STEC strains isolated from food in Germany are pathogenic for humans. Notwithstanding, the most frequent STEC serogroups in patients, except O91, were only rarely found in food.

The incongruent serogroup distributions of STEC isolates from food and from patients likely reflect the nonprobabilistic sampling schemes and differing sampling periods that underlie these populations. In addition, differences in pathogenicity among serogroups, a different serovar distribution at the serogroup level, and the fact that foodborne transmission is only 1 transmission route (*5*) should also contribute to the observed differences. Game might be a relevant, and as yet underappreciated, source for human STEC infection in Germany. Epidemiologic studies are needed to assess the risk associated with consumption of or contact with game.
